# Endothelial progenitor cells in the peripheral blood of patients with moyamoya disease labeled with superparamagnetic iron oxide *in vitro* for MRI detection

**DOI:** 10.1590/1414-431X20209974

**Published:** 2020-09-18

**Authors:** Xirui Wang, Wengao Zhang, Gangfeng Yin

**Affiliations:** Third Department of Neurosurgery, Cangzhou Central Hospital, Cangzhou, Hebei, China

**Keywords:** Endothelial progenitor cells, Superparamagnetic iron oxide, Moyamoya disease, Concentration, Label

## Abstract

Moyamoya disease (MMD) is currently thought to involve endothelial progenitor cells (EPCs). We investigated whether superparamagnetic iron oxide (SPIO) can be used to label EPCs. Mononuclear cells from 10 moyamoya disease patients were isolated, and cluster of differentiation 133 (CD133) positive cells sorted by magnetic-activated cell sorting were cultured *in vitro*. The positive rates of CD133, vascular endothelial growth factor receptor (VEGFR)-2, and cluster of differentiation 34 (CD34) were detected by flow cytometry. The cells were co-cultured with fluorescence labeled Dil-acetylated-low-density lipoprotein (Dil-ac-LDL) and Ulex europaeus agglutinin-1 (UEA-1) to observe the endocytosis of Dil-ac-LDL and binding to UEA-1. Prussian blue staining and transmission electron microscopy were used to observe the endocytosis of different SPIO concentrations in EPCs, and CCK-8 was used to detect proliferation of cells transfected with different concentrations of SPIO. T2 weighted imaging (T2WI) signals from magnetic resonance imaging after SPIO endocytosis were compared. Positive rates of CD133, VEGFR-2, and CD34 on sorted mononuclear cells were 68.2±3.8, 57.5±4.2, and 36.8±6.5%, respectively. The double-positive expression rate of CD34 and VEGFR-2 was 19.6±4.7%, and 83.1±10.4% of cells, which showed the uptake of Dil-ac-LDL and binding with UEA-1. The labeling efficiencies of SPIO at concentrations of 25 and 50 μg/mL were higher than for 12.5 μg/mL. The proliferation of cells was not influenced by SPIO concentrations of 12.5 and 25 μg/mL. After labeling, the T2WI of EPCs was reduced. The concentration of 25 μg/mL SPIO had high labeling efficiency detected by magnetic resonance imaging (MRI) without decreased EPCs viability.

## Introduction

With the development of new imaging methods, the detection rate of moyamoya disease (MMD) is increasing. New treatments have been developed for MMD, including superficial temporal artery-middle cerebral artery vascular bypass and encephalo-duro-myo-arterio-pericranial synangiosis, which have achieved better efficacy and prognosis compared with simple drug treatments (1-3). Although MMD has a high incidence of cerebral hemorrhage and cerebral ischemia, the mechanisms involved in its occurrence and development are still unclear. It was reported that the number of endothelial progenitor cells (EPCs) in the peripheral blood of patients with moyamoya disease is higher than in normal individuals, and that this increase might be relevant to the pathogenesis of MMD (4,5). In a previous study, we found that the number of EPCs at 6 months after surgery was significantly reduced compared with before surgery. In addition, cerebral vascular angiography after surgery was also reduced (6).

The development of MMD is characterized by intracranial moyamoya vascularization and blood flow change. EPCs play an important role in the occurrence and development of MMD, especially for vascularization. To investigate the pathogenesis mechanism of EPCs in moyamoya disease, a non-invasive labeling detection method was needed. Superparamagnetic iron oxide (SPIO)-labeled EPCs extracted from rabbit peripheral blood had no effect on proliferation at a concentration of 20 μg/mL (7). SPIO was previously used to label EPCs and explore the mechanism of cells involved in liver injury repair and lung cancer (8,9). EPCs in the peripheral blood of patients with MMD are very active, and their biological characteristics may differ from normal EPCs. Few studies have used SPIO labeling of EPCs from the peripheral blood of patients with MMD. In this study, we investigated the efficiency rate of SPIO labeling of EPCs isolated from the peripheral blood of patients with MMD, its influence on their biological characteristics and viability, and magnetic resonance imaging (MRI) after labeling.

## Material and Methods

### Subjects

Peripheral blood specimens were collected from 10 patients with MMD treated in the Neurosurgery Department of Cangzhou Central Hospital. All MMD patients (5 males and 5 females, aged between 32 and 53 years, mean age 41.3 years) were confirmed as early stage by cerebral angiography.

None of the patients underwent any treatment before being diagnosed. Specimens were collected in the fasting state before drug or surgical treatment. To avoid recent changes in EPCs caused by acute stroke, the study patients were selected from those without stroke attack in the last 3 months (10). Because EPCs are also influenced by vascular disease, patients with heart disease, diabetes, hyperlipidemia, and long-term smoking history were excluded (5). All eligible patients provided written informed consent before enrollment. The study was conducted in accordance with the Declaration of Helsinki and its subsequent amendments, and approved by the Institutional Review Board of Cangzhou Central Hospital (China).

### Isolation and culture of EPCs

Peripheral blood (25 mL) was collected from 10 moyamoya patients. The first 5 mL of blood withdrawn was discarded to avoid the influence of vascular endothelium injury when puncturing the vessels. The collected blood specimens were added into tubes with Ficoll-Paque (Amersham, Sweden) to isolate mononuclear cells by density gradient centrifugation. The number of CD133-positive cells in the total mononuclear cells isolated after density gradient centrifugation was detected by flow cytometry. The collected mononuclear cells were added to 300 μL buffer from the miniMACS Starting kit (Miltenyi, Germany), 100 μL FcR blocking buffer, and 100 μL CD133 magnetic beads. The cells were cultured at 4°C, resuspended with 500 μL buffer, and sorted by magnetic-activated cell sorting (miniMACS Starting Kit, Miltenyi). After the column was discharged, 1 mL of buffer was added to wash the syringe, and the labeled cells were collected.

The isolated CD133^+^ cells were seeded on a culture dish coated with fibronectin and cultured in endothelial cell growth medium-2 (EGM-2, LONZA, USA) containing 5% FBS and various inducing factors at 5% CO_2_ and 37°C. The medium was replaced every 3 days.

### Identification of EPCs

PE-labeled CD133 antibody, CD34 antibody, and VEGFR-2 antibody were used to detect CD133-positive rates after magnetic-activated cell sorting. Cells were collected after culturing for 7 days. Briefly, 10^7^ mononuclear cells were resuspended in 80 μL buffer and 20 μL FcR was added. Then, 10 μL of CD133 antibody, CD34 antibody, and VEGFR-2 antibody labeled with PE were added into three groups of cells separately. To test the CD34 and VEGFR-2 double-positive rate, PE-labeled CD34 antibody and FITC-labeled VEGFR-2 antibody were added into the fourth cell group.

All the solutions were well mixed and incubated at 4-8°C for 10 min. The cells were washed with buffer (1-2 mL/10^7^ cells) and centrifuged at 300 *g* for 10 min at 4-8°C. The supernatant was discarded and cells were resuspended with buffer and detected by flow cytometry.

Cells cultured for 7 days were co-cultured with Dil-labeled ac-LDL (acetylated low-density lipoprotein, 2.4 μg/mL, Sigma, USA) at 37°C for 2 h. The cells were fixed with 4% paraformaldehyde for 15 min and added to FITC-labeled Ulex europaeus agglutinin (UEA)-1 (10 μg/mL, Molecular Probes, USA ), incubated at 37°C for 60 min, and sealed with glycerin. The endocytosis of Dil-ac-LDL and binding of FITC-UEA-1 were observed by fluorescence microscopy. Cells that showed uptake of Dil-ac-LDL and which bound to UEA-1 were considered to be EPCs. The number of double-stained cells and the total number of cells were counted in each fluorescence microscopy field, and the probability of double-stained cells was calculated. Three fluorescence microscopy fields were chosen to calculate the mean value.

### Labeling and identification of EPCs

Resovist (28 mg Fe/mL, SPIO nanoparticle injection, Schering, Germany) was added into medium without serum to form concentrations of 0, 12.5, 25, and 50 μg/mL, and the 0 μg/mL group was set up as control. Then, media were vortexed for 60 min and mixed with Lipofectamine 2000 (Invitrogen, USA) at a ratio of 1:625 for 15 min to prepare SPIO-transfection complexes (8). After culturing for 7 days, the EPCs medium was replaced with culture medium containing SPIO-transfection medium complex at different concentrations. After 24 h, the medium was replaced with medium without iron.

The cells labeled with SPIO at different concentrations were collected and fixed with 4% paraformaldehyde for 30 min after being washed with PBS. Then, the cells were incubated for 30 min with 2% potassium ferrocyanide and 6% hydrochloric acid solution at a ratio of 1:1. The cells were completely washed with distilled water three times, re-stained for 2 min by nuclear fast red, and washed three times. The labeling efficiency was calculated.

To observe SPIO in the EPCs, the culture medium was discarded after labeling. The cells were washed with PBS three times and collected by scraping. Then, the cells were immediately placed into pre-cooled fixative, fixed with 2.5% glutaraldehyde and 1% osmium tetroxide, dehydrated by ethanol gradients, immersed in propylene oxide, embedded with Epon812, sectioned at a thickness of 50 nm, stained with uranyl acetate-lead citrate, and observed by transmission electron microscopy (TEM) (HITACHI-7500, Japan). Images were captured by a Megaview digital electron microscope photography system (Emsis, Germany).

### Proliferation detection of EPCs labeled with SPIO

CCK-8 (Beyotime Institute of Biotechnology, China) was used to detect the proliferation of EPCs. Cells were collected after digestion with 0.25% trypsin, washed with PBS once, and seeded into a 96-well plate (100 μL/well). The cells from four wells were collected every 3 days, processed using the CCK-8 kit, and incubated for 4 h. Absorbance at 490 nm was read three times and the mean value was determined to draw a growth curve.

### MRI

EPCs were digested with 2.5% trypsin/EDTA, centrifuged at 300 *g* for 10 min at 4-8°C and the supernatant discarded. After washing three times, cells at the same concentration (1×10^5^/mL) after counting were diluted with PBS into a 1.5-mL tube and examined by MRI (GE, USA) after mixing. The gray value of the T2WI of the four groups of EPCs was determined to analyze their signal intensity. The value for the control group was set at 100%, and the ratio of the other groups to the control group was used to represent their signal intensity. Each test was repeated three times to obtain the mean value.

### Statistical analysis

Statistical analysis was performed using SPSS 11.0. Data are reported as means±SD and were analyzed by one-way analysis of variance. P<0.05 indicated a statistically significant difference between values.

## Results

### Isolation and culture of EPCs

**Figure 1:**
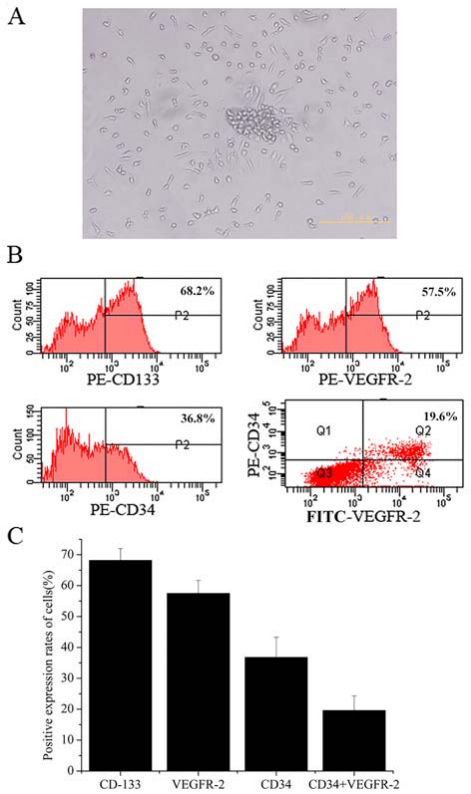
Isolation and culture of endothelial progenitor cells (EPCs). **A**, After 1 week, EPCs showed a fusiform shape and grew as a classic cell colony (scale bar 100 μm). **B**, In mononuclear cells cultured for 7 days, the positive rates of CD133, VEGFR-2, and CD34 were 68.2±3.8, 57.5±4.2, and 36.8±6.5%, respectively. The double-positive expression rate of CD34 and VEGFR-2 was 19.6±4.7%. **C**, Bar graph of panel B. Data are reported as means±SD.

As shown in Figure 1A, more than 80% of cells had a fusiform nucleus in the center and formed a classic cell colony.

**Figure 2:**
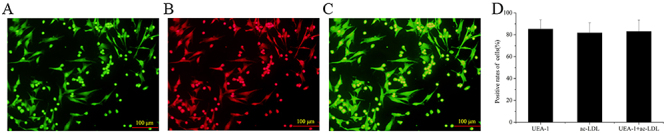
Endothelial progenitor cells were detected by immunofluorescence after co-culture with Dil-acetylated-low-density lipoprotein (Dil-ac-LDL) and FITC- Ulex europaeus agglutinin-1 (UEA-1). **A**, Cell binding with FITC-UEA-1 is shown as green. **B**, Cells that take up Dil-ac-LDL are shown as red. **C**, Cells with positive FITC-UEA-1 and Dil-ac-LDL staining (scale bar 100 μm). **D**, Bar graph of panels A, B, and C. Data are reported as means±SD.

CD133, VEGFR-2, and CD34 are commonly used to identify EPCs, and flow cytometry was used to identify the phenotype of the cells. As shown in Figure 1B, the positive rates of CD133, VEGFR-2, and CD34 were 68.2±3.8, 57.5±4.2, and 36.8±6.5%, respectively. The double-positive expression rate of CD34 and VEGFR-2 was 19.6±4.7%. To further identify the characteristics of EPCs, cells cultured for 7 days were co-cultured with Dil-ac-LDL and FITC-UEA-1 and detected by immunofluorescence. As shown in Figure 2, 83.1±10.4% of the cells showed double-positive EPCs characteristics.

### Identification of EPCs labeled with SPIO

**Figure 3:**
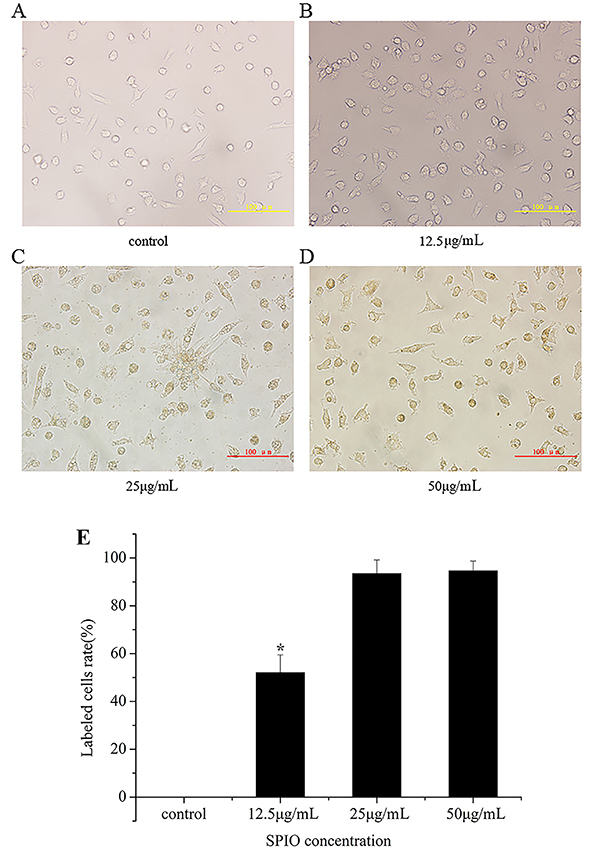
Endothelial progenitor cells were transfected with superparamagnetic iron oxide (SPIO) at different concentrations and observed by microscopy. **A**, Control group. SPIO concentrations: **B**, 12.5 μg/mL; **C**, 25 μg/mL; and **D**, 50 μg/mL (scale bar 100 μm). **E**, Bar graph of panels A, B, C, and D. Data are reported as means±SD. *P<0.05 compared to control group (ANOVA).

**Figure 4:**
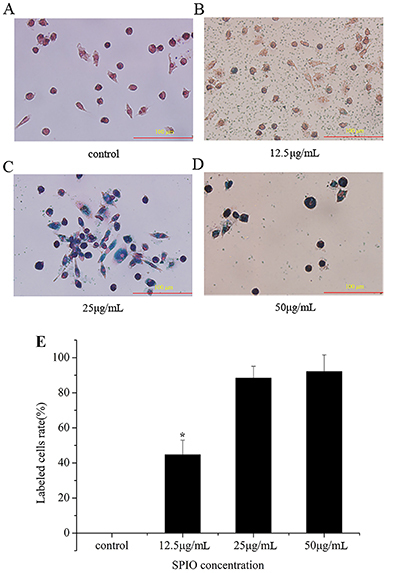
Iron levels in cells shown by staining with Prussian blue after superparamagnetic iron oxide (SPIO) transfection into endothelial progenitor cells. **A**, Control group; SPIO concentrations: **B**, 12.5 μg/mL; **C**, 25 μg/mL; and **D**, 50 μg/mL (scale bar 100 μm). **E**, Bar graph of panels A, B, C, and D. Data are reported as means±SD. *P<0.05 compared to control group (ANOVA).

**Figure 5:**
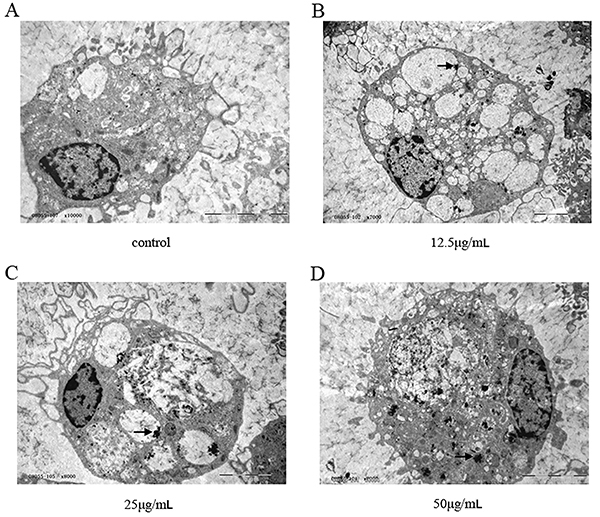
Microcosmic conditions of superparamagnetic iron oxide (SPIO) uptake by endothelial progenitor cells (after SPIO transfection at different concentrations. **A**, Control group; SPIO concentrations: **B**, 12.5 μg/mL; **C**, 25 μg/mL; and **D**, 50 μg/mL (arrow indicates SPIO granules in cells) (scale bar 5 μm).

Cells transfected with SPIO showed a pale brown color by optical microscopy, and the color was evident in the 25- and 50-μg/mL groups with the labeling efficiency 93.5±5.7 and 94.6±4.1%. The efficiency in the 12.5 μg/mL group was 52.1±7.4% (Figure 3). The iron granules in the EPCs were stained with Prussian blue. The labeling efficiency in the 12.5 μg/mL group was 44.7±8.3% and reached up to 88.4±6.7 and 92.1±9.5% in the 25 and 50 μg/mL groups (Figure 4). TEM was used to observe the microcosmic conditions of EPCs transfected with SPIO at different concentrations. As shown in Figure 5, iron granules with different degrees of aggregation were noted in the cell plasma. Therefore, SPIO at concentrations of 12.5, 25, and 50 μg/mL were successfully transfected into the cells. The labeling efficiencies of SPIO at concentrations of 25 and 50 μg/mL were significantly higher than for 12.5 μg/mL.

### Cell proliferation of EPCs labeled with SPIO

**Figure 6:**
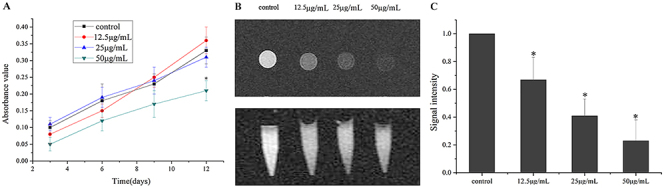
A, Cell proliferation was detected by CCK8 assay after endothelial progenitor cells (EPCs) were transfected with superparamagnetic iron oxide (SPIO) at different concentrations. **B**, Magnetic resonance plain scan imaging of EPCs transfected with SPIO at different concentrations. **C**, Gray value comparison of T2 weighted imaging of EPCs transfected with SPIO at different concentrations. Data are reported as means±SD (ANOVA). *P<0.05, compared to control group.

Compared with the control group with no SPIO, the proliferation of EPCs in the 50-μg/mL group was significantly inhibited (P<0.05), but not in the 12.5 and 25-μg/mL groups (P>0.05). This indicated that SPIO at a concentration of 50 μg/mL, but not 12.5 and 25 μg/mL, had high cytotoxicity (Figure 6A).

### *In vitro* MRI of EPCs labeled with SPIO

Figure 6B shows the MRI of EPCs labeled with SPIO of different concentrations. The T2WI signal intensity of cells labeled with SPIO was significantly reduced compared with the control group with no SPIO (P<0.05). A higher SPIO concentration led to a lower signal intensity of T2WI, and the T2WI signal intensity of the 25 μg/mL group was significant compared with the control group with no SPIO (Figure 6C). Data in Figure 6A, B, and C show that a SPIO concentration of 25 μg/mL did not inhibit the proliferation of EPCs, and that the low T2WI signal intensity was more evident for easy testing.

## Discussion

EPCs are important for neonatal vascularization because they produce and secrete growth factors (11,12). The occurrence and development of MMD is accompanied by angiogenesis. Although its pathogenesis is unclear, it is thought that abnormalities of EPCs are involved (13). Therefore, the investigation of EPCs might help clarify the mechanism of MMD. Currently, imaging methods including MRI cannot identify EPCs *in vivo*; however, labeling technology and imaging systems can be used to trace the cells.

High-accuracy extraction of EPCs is required for successful cell labeling. Differential adhesion culture methods are often used to obtain EPCs from mononuclear cells. However, the purity of EPCs is usually low after differential adhesion culture. To obtain highly purified EPCs, we used magnetic-activated cell sorting. CD133, CD34, and VEGFR-2 are considered characteristic surface antigens of EPCs. CD34^+^VEGFR-2^+^ EPCs express CD133, which is not expressed by mature endothelial cells and monocytes, and therefore can be used as a specific marker for cell sorting (14,15). We used magnetic-activated cell sorting by CD133 to obtain highly purified EPCs from mononuclear cells.

SPIO is a new MRI contrast agent with a significant T2 negative enhancement effect. Currently, the use of SPIO to label EPCs has been demonstrated in animal models (16). However, SPIO is cytotoxic, which can influence the proliferation and adhesion ability of cells. SPIO toxicity is caused by iron, an essential element for metabolism in normal cells. The moderate accumulation of iron enhances the proliferation of cells, but excessive iron is cytotoxic (17-20). Therefore, it is necessary to select an appropriate SPIO concentration when labeling EPCs. Arbab et al. and Himes et al. (21,22) reported that a concentration of SPIO lower than 50 μg/mL was not significantly cytotoxic for EPCs. Frank et al. and van den Bos et al. (23,24) reported that the transfection reagent significantly improved the labeling efficiency of SPIO and that the cytotoxicity of low concentrations of transfection reagent was not significant. To improve the labeling efficiency, SPIO was used to label the cells assisted by a transfection reagent. In our study, the labeling efficiency was high at 25 and 50 μg/mL. However, SPIO at 50 μg/mL had a significant inhibitory effect on the proliferation of EPCs compared with the 12.5 and 25 μg/mL concentrations. Thus, 25 μg/mL achieved highly efficient labeling without influencing cell viability, indicating this concentration was appropriate for the labeling of EPCs. Previous studies reported that 20 μg/mL SPIO was used to label EPCs in rabbit peripheral blood and 50 μg/mL was required for mice (7,8), suggesting different SPIO concentrations might be required for labeling in different species. A concentration of 7 μg/mL alkyl-polyethylenimine 2 kDa (PEI2K) stabilized SPIO for the labeling of EPCs in a mouse lung carcinoma xenograft model (9), and a higher concentration might increase the potential for cytotoxicity. Furthermore, the activity of EPCs was not affected when the SPIO concentration was less than 70 μg/mL in the peripheral blood of healthy minipigs (25). However, this study indicated that 50 μg/mL SPIO had a significant inhibitory effect on the proliferation of MMD human peripheral blood EPCs.

When using SPIO labeling, it is important to distinguish non-labeled cells from labeled cells. SPIO granules are small with a long half-life, which has a significant T1WI relaxation effect. The T1WI relaxation effect was significantly reduced during T2WI relaxation. Concurrent with the increase in T1WI signal, T2WI tissue signals were significantly reduced (26). We also found that the T2WI signal and signal intensity of EPCs labeled with SPIO at 25 μg/mL was significantly reduced compared with the control group. Thus, SPIO at a concentration of 25 μg/mL successfully labeled the EPCs in this study.

In summary, SPIO was a useful marker for endothelial progenitor cells in the peripheral blood of patients with MMD. The labeling efficiency of SPIO at a concentration of 25 μg/mL on endothelial progenitor cells was high with low cytotoxicity, and provided clear MRI. This study reports a new strategy to determine the role of changes in EPCs in MMD pathogenesis.
